# Genome-wide mapping of complement system proteins for islet autoimmunity in the DAISY and TEDDY children

**DOI:** 10.21203/rs.3.rs-5975824/v1

**Published:** 2025-02-10

**Authors:** Xiaowei Hu, Bobbie-Jo M Webb-Robertson, Hemang M Parikh, Ernesto S Nakayasu, Suna Onengut-Gumuscu, Wei-Min Chen, Ashley Frazer-Abel, Thomas O Metz, Stephen S Rich, Marian J Rewers, Ani Manichaikul

**Affiliations:** Department of Genome Sciences, University of Virginia; Biological Sciences Division, Pacific Northwest National Laboratory; Health Informatics Institute, Morsani College of Medicine, University of South Florida; Biological Sciences Division, Pacific Northwest National Laboratory; Department of Genome Sciences, University of Virginia; Department of Genome Sciences, University of Virginia; Exsera BioLabs, Division of Rheumatology, Department of Medicine, University of Colorado Anschutz Medical Campus; Biological Sciences Division, Pacific Northwest National Laboratory; Department of Genome Sciences, University of Virginia; Barbara Davis Center for Diabetes, School of Medicine, University of Colorado Anschutz Medical Campus; Department of Genome Sciences, University of Virginia

**Keywords:** islet autoimmunity, T1D, complement system proteins, protein quantitative trait locus (pQTL), DAISY, TEDDY

## Abstract

**Background:**

Type 1 diabetes (T1D) is characterized by the autoimmune destruction of the insulin-producing beta cells, and there is no cure yet for the disease. While islet autoantibodies are well-recognized biomarkers that mark the onset of islet autoimmunity (IA) and are predictors of T1D, few additional biomarkers are available to monitor disease progression. Recent studies have reported the involvement of complement system proteins in the initiation and progression of IA in the study of T1D. However, the genetic factors of complement system proteins at the time of triggering of IA is unknown.

**Results:**

Through complement system protein quantitative trait locus (pQTL) mapping analysis of 170 participants from the Diabetes Autoimmunity Study in the Young (DAISY), we identified 240 statistically significant pQTLs (false discovery rate, FDR < 0.1) from pooled and IA case-stratified analyses. Replication analysis conducted on 385 IA cases from The Environment Determinants of Diabetes in the Young (TEDDY) study confirmed 68 significant (FDR < 0.05) pQTLs in total for C8A, C8B, CFB, C4A, and MBL2. Furthermore, all replicated pQTLs of CFB and C4A were previously reported to be associated with T1D risk.

**Conclusions:**

We identified and replicated 68 pQTLs for five complement system proteins (C8A, C8B, CFB, C4A, and MBL2) in the young population. Among them, all replicated pQTLs of CFB and C4A are also associated with T1D risk. Our study provides evidence of complement system proteins as potential protein biomarkers underlying the development and progression of T1D.

## Background

Type 1 diabetes (T1D) is a complex autoimmune disease where an autoimmune attack destroys the pancreatic islet beta cells, which eventually results in a complete dependency on exogenic insulin. There is no cure for T1D yet, and the disease progression is not well understood. Hence, the development of biomarkers that can help track disease progression and determine disease causes is important for T1D prevention and therapy. The islet antibodies are well-recognized and reliable diagnostic biomarkers that mark the onset of islet autoimmunity (IA), and persistent multi-positivity of islet autoantibodies can be used to predict individuals at high risk of developing T1D^1,2^. However, the genetic mechanisms of the initiation of IA, as well as the progression from IA to clinical diabetes, are still largely unknown.

The complement system, also known as complement cascade, plays an important role in the innate immune system, which helps to fight bacterial or viral infections and promotes clearance of damaged cells^3^. Three interrelated pathways activate the complement system, specifically the classical, lectin, and alternative pathways. These activation pathways converge at the terminal pathway, where much of the effector function resides^3^. The complement system also modulates adaptive immunity through activation by antibodies, including autoantibodies^4^. Several studies have suggested that the dysregulation of the complement system contributes to the pathogenesis of autoimmune diseases, such as systemic lupus erythematosus^5^, rheumatoid arthritis^6^, and T1D^7,8^. More specifically, increasing studies have reported the involvement of the complement system in the initiation and progression of IA for the study of T1D^9,10^. Furthermore, recent studies observed lower levels of several complement system proteins in children with islet autoantibodies compared to healthy controls^11–14^. However, the genetic factors that result in decreased abundance of complement system proteins at the time of triggering of IA are currently unclear^15^.

Circulating plasma proteins play a fundamental role in human biological processes and are frequently the targets of pharmaceutical interventions^16^. Protein quantitative trait locus (pQTL) mapping is an approach that can identify genetic variants underlying variation of protein expression levels. In prior studies, pQTL analysis has led to critical advances in knowledge of the genetic architecture of plasma proteins and their relationship to disease^17–21^.

In this study, we aim to improve our understanding of the genetic factors of complement system proteins at the time of triggering of IA and bring us closer to dissecting the genetic mechanisms underlying the development and progression of T1D. Here, we performed pQTL analysis of 170 participants in the Diabetes Autoimmunity Study in the Young (DAISY) to identify statistically significant pQTLs for complement system proteins. Complement system proteins for DAISY participants were measured by two different protein assay platforms, selected reaction monitoring (SRM)^22^, and Exsera Biolabs^23^ (hereinafter referred to as Exsera). Complement system proteins measured by two platforms were approximately overlapped in half. Hence, using proteins from both platforms provides a more comprehensive list of complement system proteins for study. Replication analysis was then conducted on 385 IA cases in the Environment Determinants of Diabetes in the Young (TEDDY) study and the proteins were measured by SRM for TEDDY participants. Finally, we used T1D Knowledge Portal to examine replicated pQTLs for their association with T1D and other disease-relevant traits.

## Results

### Participant characteristics

The demographic and clinical characteristics of study samples are summarized in Table 1, which includes 170 samples with 131 IA cases and 39 controls for DAISY discovery analysis, and 385 IA cases for TEDDY replication analysis after combining available proteomics and genotype data (Methods). Overall, the participants in the DAISY study had a median age of 6.2 years which was older than TEDDY participants with a median age of 1.8 years (Table 1). The percentage of first-degree relatives with T1D in DAISY was 54%, much higher than 22% from TEDDY (Table 1). For the other two clinical characteristics, female (%) and self-reported Non-Hispanic White (NHW) (%), the percentages were similar between the two studies. Females accounted for 46% and 44% in DAISY and TEDDY, respectively, and NHW was the most common population in both studies.

### *cis*-pQTLs discovery analysis in DAISY

We first performed *cis*-pQTL mapping for DAISY samples in pooled (IA cases and controls) and IA case-stratified analyses, respectively (Methods). There were 19 and 16 complement system proteins measured by the SRM and Exsera platform, respectively, for DAISY samples, and 10 proteins overlapped between the two platforms (Table 2). We observed 14 proteins with false discovery rate (FDR)-significant (FDR<0.1) *cis*-pQTLs (Table 2). MBL2 has the greatest number of FDR-significant *cis*-pQTLs with 264 and 211 *cis*-pQTLs identified from Exsera pooled and case-stratified analysis, respectively (Additional file 2: **Table S1**). However, there were no statistically significant *cis*-pQTLs of MBL2 from the SRM platform. Additionally, MBL2 had the *cis*-pQTLs with the greatest strength of statistical significance (chr10:52771475-T, chr10:52773600-G, chr10:52775077-T with p-value=3.12×10^−19^ and p-value=5.22×10^−16^ from Exsera pooled and case-stratified analysis, respectively, Additional file 2: **Table S1**). Among other genes, CFH and C4B had FDR-significant *cis*-pQTLs from both SRM and Exsera platforms (Table 2).

For six proteins that had many FDR-significant *cis*-pQTLs (i.e., CFH, C8A, CFB, C4A, C4B, and MBL2), we performed fine-mapping to identify potential causal *cis*-pQTLs for each protein (Methods). For the proteins C8A, CFB and C4B, we observed corresponding 95% credible sets (CS) containing 29, 26, and 9 fine-mapped variants, respectively, from the SRM pooled analysis. For CFH, we observed a single 95% CS with 69 fine-mapped variants in the pooled analysis of protein levels from the Exsera platform (Additional file 1: **Fig. S1–4**). MBL2 has fine-mapped variants from both Exsera analyses, where 14 variants were identified in two 95% CSs from pooled analysis, and 4 variants were identified in one 95% CS from case-stratified analysis (Additional file 1: **Fig. S5**). However, there were no fine-mapped variants for CFH and C4A from SRM pooled analysis (Additional file 2: **Table S1**). We used non-fine-mapped *cis*-pQTLs for these two proteins from the corresponding analysis. After fine-mapping analysis, we identified 240 FDR-significant (FDR<0.1) *cis*-pQTLs in total from DAISY discovery analysis.

### Replicated *cis*-pQTLs in TEDDY

To follow up on our discovery pQTL analysis in DAISY, we performed replication analysis in TEDDY for the 240 *cis*-pQTLs reported in Additional file 2: **Table S1**, which maps to 14 proteins. A *cis*-pQTL was deemed replicated if it had FDR-corrected p-value< 0.05. Of these, we observed 68 *cis*-pQTLs passing the replication threshold, including 27 for C8A, 23 for CFB, 12 for C4A, 4 for MBL2, and 2 for C8B, respectively. The summary of all replicated *cis*-pQTLs for C8B and MBL2, and top replicated *cis*-pQTLs for C8A, CFB and C4A is presented in Table 3. The other replicated *cis*-pQTLs for C8A, CFB and C4A are presented in Additional file 3: **Table S2**.The replicated *cis*-pQTLs of CFB, C4A, and MBL2 decreased protein expression levels, while the replicated *cis*-pQTLs of C8A and C8B increased protein levels.All replicated *cis*-pQTLs had the same direction of effect between DAISY and TEDDY. However, the replicated signals in TEDDY generally achieved a lower level of statistical significance compared to DAISY. One exception to this finding was the CFB pQTL (chr10:31562639-A: Beta (SE)=−0.45 (0.11) and p-value=3.92×10^−5^ in TEDDY vs. Beta (SE)=−0.34 (0.08) and p-value=4.37×10^−5^ in DAISY; Additional file 2: **Table S1**;Additional file 3: **Table S2**).

### Examination of association with T1D for replicated pQTL variants

We further examined the association of replicated *cis*-pQTLs with T1D through publicly available resources. The T1D Knowledge Portal (https://t1d.hugeamp.org) enables searching of human genomic variants linked to T1D and other related phenotypes. We used the T1D Knowledge Portal and found that all 23 and 12 replicated *cis*-pQTLs for CFB and C4A respectively have been reported genome-wide significantly (p-value<5×10^−8^) associated with T1D risk (Additional file 3: **Table S2**; Additional file 1: **Fig. S6–7**). The T1D association p-values were obtained from a previous T1D study^24^, and the p-values range from 1.11×10^−37^ (C4A: rs9262570) to 6.29×10^−235^ (CFB: rs114355928) (Additional file 3: **Table S2**). For the other replicated *cis*-pQTLs, we found that they were significantly associated with highlight scatter reticulocyte count (C8A and C8B, Additional file 1: **Fig. S8–9**), chronic kidney disease and reticulocyte count (MBL2, Additional file 1: **Fig. S10–11**).

## Discussion

Previous reports have examined the involvement of the complement system in the initiation and progression of IA for the study of T1D^8,11,12,14^, however, the genetic factors underlying the complement system proteins at the time of triggering of IA are poorly unknown. Our study investigated genetic variants that are associated with complement system proteins in DAISY, a prospective cohort of children from the general population who either had a first degree relative with T1D or had a high-risk human leukocyte antigen (HLA) genotype. From pooled and IA case-stratified analyses with 170 participants in the DAISY, 240 significant (FDR < 0.1) *cis*-pQTLs were associated with 14 complement system proteins. We replicated 68 *cis*-pQTLs with statistical significance (FDR < 0.05) in the 385 IA cases from the TEDDY study. The 68 replicated *cis*-pQTLs represent C8A and C8B, CFB and C4A (within the HLA region), and MBL2. The CFB and C4A *cis*-pQTLs are known genetic associations with T1D. However, the strong linkage disequilibrium within the region has limited our understanding of the independent contribution of complement system to T1D risk. Our study provides new insight into the role of complement system on the disease progression.

CFB (Complement Factor B) is located on chromosome 6 and between the HLA class II and class I regions^7^. The HLA region is the single most important genetic determinant of T1D susceptibility. The variability in the HLA region has been estimated to explain approximately 60% of the genetic influence of T1D^25^. CFB is a component of the alternative pathway of complement activation. The detailed function of CFB has been reported in a previous study^15^. T1D-associated genetic variants in CFB have been reported in European ancestry^26,27^ and Northern India^28^. The 23 replicated *cis*-pQTLs of CFB identified in our study, which decrease protein level, are found to be significantly associated with increasing T1D risk in a large-scale genomic study of T1D^24^. The T1D association p-value ranges from 5.38×10^− 43^ (rs115272033, *EHMT2* intron) to 6.29×10^− 235^ (rs114355928, *TSBP1* intron) (Additional file 3: **Table S2**).

C4A (Complement Component 4A) is also located between the HLA class II and class I regions^7^ and is part of the classical and lectin pathways of activating complement system. Two functionally distinct genes, C4A and C4B, code the C4 protein together. Limited T1D association studies have been focused on the structural variation of this region^15^. The previously reported associations of genetic variants in C4A with T1D focused on European ancestry population^29,30^. Our study found that the 12 replicated *cis*-pQTLs of C4A were previously identified to be significantly associated with increasing T1D risk^24^. The T1D association p-value ranges from 1.11×10^− 37^ (rs9262570, intergenic) to 1.33×10^− 88^ (rs9263822, *PSORS1C3* intron) (Additional file 3: **Table S2**).

Our study identified three genes outside of HLA region (C8A, C8B and MBL) in addition to two genes in the HLA region. Both C8A (Complement C8 Alpha Chain) and C8B (Complement C8 Beta Chain) are located on chromosome 1 and encode Complement Component 8 (C8) protein. C8 participates in the formation of the membrane attack complex, which causes cell lysis and/or pro-inflammatory signaling^15^.

MBL2 (Mannose-Binding Lectin 2) is located on chromosome 10 and is the only single gene to encode human Mannose-Binding Lectin (MBL). MBL is a soluble lectin that activates the lectin complement pathway by recognizing microorganisms through the carbohydrate-recognition domain, thereby modulating inflammation^31^. Although our replicated *cis*-pQTLs of MBL2 were not associated with T1D, previous studies have identified their associations with type 2 diabetes and pneumonia. For example, the variant rs1800450 has been linked to type 2 diabetes in diverse populations, including the full-heritage Pima Indians and the Old Order Amish^32^, and the North Chinese Han population^33^. Additionally, Uysalol et al.^34^ reported that the rs1800450 genotypes associated with low MBL expression were significantly more common in patients with pneumonia and severe infections.

Strengths of this study include being one of the very few studies that examined pQTL mapping of complement system proteins in children with high risk of developing T1D and the use of independent study data to replicate significant pQTL signals. However, some limitations warrant mentioning. First, the statistical power of identifying significant *cis*-pQTLs in our study is still limited due to the sample size of both discovery and replication analyses. Second, the protein assay platform in the replication analysis is different from that in the discovery analysis for the significant findings of MBL2. We only identified statistically significant *cis*-pQTLs in the Exsera platform for MBL2 in the DAISY discovery analysis, however, the replication analysis in TEDDY was only available to use measured protein levels from a different protein assay, the SRM platform. Hence, future validation for the identified *cis*-pQTLs of MBL2 needs to be conducted on measured protein levels from the Exsera platform. Lastly, the participants in our study are predominantly composed of Non-Hispanic Whites. Hence, our findings cannot be generalized to non-European ancestry populations yet. Future research focusing on non-European or diverse ancestries will help improve the understanding of genetic mechanisms underlying the complement system and provide a more comprehensive insight into the role of complement system proteins in the etiology of T1D.

## Conclusions

In summary, this study performed pQTL mapping of complement system proteins in children at high risk of developing T1D and replicated significant signals in an independent study of children with T1D. Our study confirmed that genetic variants regulating two complement system proteins CFB and C4A, located in the HLA-region, are associated with an increased risk of T1D. Further, we identified three non-HLA-region complement system proteins (C8A, C8B, and MBL2) as potential biomarkers with a putative role in the etiology of T1D.

## Methods

### Overview of approach

We performed *cis*-pQTL mapping for 170 samples (131 IA cases and 39 controls) in The Diabetes Autoimmunity Study in the Young (DAISY) as discovery analysis to identify statistically significant *cis*-pQTL variants for complement system proteins. To follow up on our pQTL analysis in DAISY, we conducted replication analysis for 385 IA samples from The Environmental Determinants of Diabetes in the Young (TEDDY) study. Finally, we used the T1D Knowledge Portal (https://t1d.hugeamp.org/) to examine replicated *cis*-pQTLs for their association with T1D and other disease-relevant traits.

### DAISY: study design, proteomic profiling and genotyping

Study design: DAISY is a prospective cohort of 2,547 children from the general population who either had a first degree relative with T1D or had a high-risk human leukocyte antigen (HLA) genotype. The participants were recruited in Denver, Colorado between 1993 and 2004, and followed for up to 21 years^35,36^. Follow-up results are available through April 4, 2022. Written informed consent was obtained from participants and parents. The Colorado Multiple Institutional Review Board approved all protocols. The primary goal of DAISY is to learn how genes and the environment interact to cause childhood T1D^35^.

Proteomic profiling: The peptides of complement system proteins were measured by two different assay platforms for DAISY participants, selected reaction monitoring (SRM)-based^22^, and Exsera Biolabs-based (referred herein as Exsera)^23^. The Exsera targeted proteomics use commercial immunoassays in a College of American Pathologists/Clinical Laboratory Improvement Amendments (CAP/CLIA)-accredited laboratory. The details of two protein assay platforms for DAISY participants are described in a previous study^14^.

Genotype quality control and imputation: Genome-wide genotyping was performed using the custom designed Infinium TEDDY-T1D Exome array (Illumina) and was genotyped at the University of Virginia (UVA) Genome Sciences Laboratory following the manufacturer’s protocol (Illumina). The following quality control (QC) criteria were applied: 1) samples with a genotype call rate < 0.95 were removed; and 2) single nucleotide polymorphism (SNP) level QC included removal of monomorphic SNPs, and removal of SNPs that deviated from Hardy-Weiberg equilibrium (p-value < 1×10^− 20^ at the HLA region or p-value < 1×10^− 6^ otherwise). Genome-wide imputation used the Trans-Omics for Precision Medicine (TOPMed) multi-ancestry reference panel (version R2). The SNP was selected if minor allele frequency (MAF) ≥ 0.05 and imputation quality R^2^ ≥ 0.7. The details of QC criteria of genomic variants used in this study are provided in a previous study^37^.

### TEDDY: study design, proteomic profiling and genotyping

Study design: TEDDY is an international prospective study that was designed to identify T1D-associated environmental factors in children who carry a high genetic risk for the disease^38^. The participating clinical centers include Colorado, Georgia/Florida, and Washington in the US, and Finland, Germany, and Sweden in Europe. We only reported summary statistics of self-reported Non-Hispanic White (NHW) from US centers for TEDDY as many missing or unknown race/ethnicity were reported from European centers. For TEDDY study, all procedures were approved by the ethics committees / institutional review boards including Colorado Multiple Institutional Review Board (04–0361); Medical College of Georgia Human Assurance Committee (2004–2010)/Georgia Health Sciences University Human Assurance Committee (2011–2012)/Georgia Regents University Institutional Review Board (2013–2017)/Augusta University Institutional Review Board (2017-present) (HAC 0405380); University of Florida Health Center Institutional Review Board (IRB201600277); Washington State Institutional Review Board (2004–2012)/Western Institutional Review Board (2013–present) (20130211); Ethics Committee of the Hospital District of Southwest Finland (Dnro168/2004); Bayerischen Landesärztekammer (Bavarian Medical Association) Ethics Committee (04089); and Regional Ethics Board in Lund, Section 2 (2004–2012)/Lund University Committee for Continuing Ethical Review (2013-present) (217/2004). In addition, TEDDY is monitored by an external evaluation committee formed by the National Institutes of Health, Bethesda, MD, U.S.A.

Proteomic profiling and genotyping: Both proteomics and imputed genotype data were provided by the TEDDY Data Coordinating Center. The peptides of complement system proteins for TEDDY participants were measured by SRM-based assay. The details of the protein assay for TEDDY are described in a previous study^13^. SNPs were genotyped using the ImmunoChip and/or the TEDDY-T1DexomeChip at the Center for Public Health Genomics at the UVA, US. GWAS imputation analysis was conducted using the TOPMed Version R2 (built from 97,256 deeply sequenced human genomes containing 308,107,085 genetic variants), the 1000 Genomes, and a subset of the TEDDY subjects (n = 1,119) with the whole-genome sequencing data as reference panels. MetaMinimac2 was used to combine genotype data imputed against these three reference panels. For imputed genotype data, we retained rare variants with MAF > 0.05 in unrelated controls with European ancestry and with imputation quality R^2^ > 0.50.

### cis -pQTL mapping in DAISY

A *cis*-pQTL mapping is to test the association between measured protein levels and *cis*-pQTL genomic variants via statistical model. We defined a *cis*-pQTL genomic variant as a SNP within +/− 1Mb of the transcription start site (TSS) of the corresponding protein-coding gene. We applied a linear mixed model adjusted for age, sex, self-reported race/ethnicity, protein plate effects, first-degree relative with T1D (yes or no), the first two principal components (PCs) of genetic ancestry, and genetic relationship matrix (GRM) to perform *cis*-pQTL mapping in our study. The association analyses were conducted using R/GENESIS^39^. The mapping results were then filtered on 1) expected heterozygosity count (EHC) > 6 for WGS data, and 2) imputation quality > 0.3 and EHC > 6 for imputation genotypes by using R/EasyQC^40^.

We first performed *cis*-pQTL mapping on DAISY participants with Islet autoimmunity (IA), referred herein as IA cases. Participants were considered to have IA if they were positive for one or more islet autoantibodies tests on two or more consecutive visits or being autoantibody positive with a diagnosis of diabetes at the next visit by the American Diabetes Association criteria^41^. For protein levels of IA cases, 1) we averaged measured peptides if a participant had multiple peptides at one time point, and 2) we used the earliest time point if a participant had multiple visits for peptide measures. We then applied log2-transformation on the selected peptides and treated the transformed peptides as outcome variables for the linear mixed model. Due to limited IA cases in DAISY, we also conducted case-control pooled analysis for DAISY with additional group adjustment (i.e., case or control) to increase statistical power of *cis*-pQTL mapping. For protein levels of controls, we followed the same procedure as IA cases except for the selection of time points for controls who have multiple peptides by different visits. To obtain a similar age as IA cases for most controls, we selected the closest time point to the median age of selected visits of IA cases for controls. Finally, we applied false discovery rate (FDR) correction (Benjamini-Hochberg) at 10% on *cis*-pQTL mapping results to identify statistically significant *cis*-pQTLs in DAISY by different protein assay platforms and by different stratified analyses respectively. For proteins with many FDR-significant *cis*-pQTLs, we further performed fine-mapping using SuSiE^42^ to identify potential causal *cis*-pQTLs. The variant clustered in a 95% credible set (CS) is considered as a fine-mapped variant^42^.

### Replication analysis in TEDDY

To follow-up on statistically significant *cis*-pQTLs identified in DAISY discovery analysis, we applied a linear mixed model adjusted for age, sex, protein plate effects, clinical centers, first-degree relative with T1D (yes or no), the first two PCs of ancestry, and GRM to test association between DAISY FDR-significant *cis*-pQTLs and log2-transformed peptides for TEDDY IA cases. We then applied FDR correction at 5% on TEDDY mapping results to identify replicated *cis*-pQTLs.

### PheWAS of replicated cis-pQTLs

The T1D Knowledge Portal (https://t1d.hugeamp.org) enables searching of human genomic variants linked to T1D and other related phenotypes. We used T1D Knowledge Portal to examine replicated *cis*-pQTLs for their association with T1D and other disease-relevant traits.

## Figures and Tables

**Table 1:** Clinical characteristics of samples in DAISY and TEDDY.

	DAISY	TEDDY
Sample size	170 (IA cases=131, controls=39)	385 IA cases
Age^#^ (year)	6.2±3.8	1.8±1.2
Female (%)	78 (46%)	168 (44%)
Self-reported NHW (%)	166 (98%)	106 (89%)*
First-degree relative with T1D: yes (%)	91 (54%)	84 (22%)

median ± standard deviation; DAISY, the Diabetes Autoimmunity Study in the Young study; TEDDY, The Environment Determinants of Diabetes in the Young study; IA, islet autoimmunity

# age matches with sample’s protein collection time; T1D, type 1 diabetes; NHW, Non-Hispanic White

* TEDDY participants are from three United States (US) clinical centers (Colorado, Georgia/Florida, Washington) and three European clinical centers (Finland, Germany, Sweden), the summary statistics are from three US clinical centers as European clinical centers report many missing or unknown race/ethnicity.

**Table 2:** Summary of FDR-significant (FDR<0.1) *cis*-pQTLs identified in DAISY.

		SRM			Exsera		
		No. Significant *cis*-pQTLs			No. Significant *cis*-pQTLs		

Protein	Chr	Pooled	Case-stratified	Total	Pooled	Case-stratified	Total
**C1QC**	1	0	0	0	3	2	5
**CFH**	1	50	0	50	69	0	69
C8A	1	29	0	29	--	--	--
C8B	1	2	0	2	--	--	--
**CFI**	4	0	0	0	0	3	3
C6	5	3	0	3	--	--	--
C7	5	0	0	0	--	--	--
C9	5	0	0	0	--	--	--
**CFB**	6	26	0	26	0	0	0
**C2**	6	0	0	0	0	5	5
C4	6	--	--	--	0	0	0
C4A	6	17	0	17	--	--	--
**C4B**	6	9	0	9	0	1	1
**C5**	9	0	0	0	0	0	0
C5A	9	--	--	--	5	3	5
C5B	9	--	--	--	0	3	3
C8G	9	0	0	0	--	--	--
**MBL2**	10	0	0	0	14	4	14
C1R	12	0	0	0	--	--	--
C1S	12	0	0	0	--	--	--
CFD	19	--	--	--	0	0	0
C3	19	0	0	0	0	0	0
C3A	19	--	--	--	0	0	0
C3B	19	--	--	--	0	1	1
**CFP**	X	0	0	0	0	0	0

DAISY, the Diabetes Autoimmunity Study in the Young study; 10 overlapped proteins between SRM and Exsera are highlighted in bold; SRM, selected reaction monitoring mass spectrometry assay; FDR, false discovery rate; Total, union of significant *cis*-pQTLs after fine mapping from pooled and case-stratified analyses; –, protein data was not available in the corresponding assay.

**Table 3:** Summary of replicated *cis*-pQTLs in TEDDY.

	DAISY	TEDDY
Sample size	170 (IA cases=131, controls=39)	385 IA cases
Age^#^ (year)	6.2±3.8	1.8±1.2
Female (%)	78 (46%)	168 (44%)
Self-reported NHW (%)	166 (98%)	106 (89%) *
First-degree relative with T1D: yes (%)	91 (54%)	84 (22%)

Chr, chromosome; FDR, false discovery rate; Beta (SE)/P-value, *cis*-pQTL mapping effect size (standard error)/P-value for 385 TEDDY IA cases; TEDDY, The Environment Determinants of Diabetes in the Young study; IA, islet autoimmunity; T1D, type 1 diabetes

* top pQTL variant, see Supplementary Table S2 for full replicated *cis*-pQTL list.

## Data Availability

The TEDDY Immunochip (SNP) data that support the findings of this study have been deposited in NCBI’s database of Genotypes and Phenotypes (dbGaP) with the primary accession code phs001442.v4.p3. The TEDDY-T1DExome Array data that support the findings of this study have been deposited in NCBI’s database of Genotypes and Phenotypes (dbGaP) with the primary accession code phs001442.v4.p3. The TEDDY Whole Genome Sequencing (WGS) data that support the findings of this study have been deposited in NCBI’s database of Genotypes and Phenotypes (dbGaP) with the primary accession code phs001442.v4.p3. The TEDDY Proteomics Discovery Phase data that support the findings of this study are available from the Mass Spectrometry Interactive Virtual Environment (MassIVE) repository https://massive.ucsd.edu/ via its dataset identifier MSV000091560 (https://doi.org/doi:10.25345/C5V11VW3F). The TEDDY Proteomics Validation Phase data that support the findings of this study are available from the Mass Spectrometry Interactive Virtual Environment (MassIVE) repository https://massive.ucsd.edu/ via its dataset identifier MSV000091562 (https://doi.org/doi:10.25345/C5KH0F84V).
